# Life history and description of larva and pupa of *Platyphileurus felscheanus* Ohaus, 1910, a scarabaeid feeding on bromeliad tissues in Brazil, to be excluded from Phileurini (Coleoptera, Scarabaeidae, Dynastinae)

**DOI:** 10.3897/zookeys.389.6888

**Published:** 2014-03-14

**Authors:** Fabiano F. Albertoni, Frank-Thorsten Krell, Josefina Steiner, Anne Zillikens

**Affiliations:** 1 Departamento de Biologia Celular, Embriologia e Genética, Centro de Ciências Biológicas, Universidade Federal de Santa Catarina, Campus Universitário Trindade, 88.040-900, Florianópolis, SC, Brazil; 2 Entomologia, Museu de Zoologia da Universidade de São Paulo, São Paulo, SP, Brazil; 3 Department of Zoology, Denver Museum of Nature & Science, 2001 Colorado Boulevard, Denver, Colorado 80205-5798, U.S.A.; 4 Medizinisch-Naturwissenschaftliches Forschungszentrum, Eberhard Karls Universität Tübingen, Ob dem Himmelreich 7, 72074 Tübingen, Germany

**Keywords:** *Surutu jelineki*, Cyclocephalini, Bromeliaceae, beetle, third instar, dynastine tribe, classification

## Abstract

The third instar larvae and the pupae of *Platyphileurus felscheanus* Ohaus, 1910 (Phileurini), recently synonymized with *Surutu jelineki* Endrődi, 1975 (Cyclocephalini), are described and illustrated, and some life history information is given. The larvae were collected and reared in bromeliads in rain forests of Santa Catarina state in southern Brazil. The systematic position of this monotypic genus is reassessed at the tribe level by considering larval and adult morphological characters. Both character sets, being described and illustrated, suggest the placement of *Platyphileurus* in the tribe Oryctini.

## Introduction

### The enigmatic *Platyphileurus felscheanus* / *Surutu jelineki*

The rhinoceros beetle *Platyphileurus felscheanus* Ohaus, 1910 (Scarabaeidae: Dynastinae) was described twice as a new species, first under this name in the tribe Phileurini and 65 years later as *Surutu jelineki* by Endrődi (1975) in the tribe Cyclocephalini. Grossi et al. (2010) recently synonymized those two names.

The monotypic genus *Platyphileurus* Ohaus, 1910 is known only from Brazil (Grossi et al. 2010) with one erroneous record from the Ilha do Principe (West Africa) (Endrődi 1977). Grossi et al. (2010) extended the known distribution of the species from Santa Catarina (Ohaus 1910) and Rio de Janeiro (Endrődi 1985) to the states of Bahia, Espírito Santo, Minas Gerais and Paraná.

*Platyphileurus felscheanus* can be recognized by its flat body, especially anteriorly, by the elytra being laterally dilated posteriorly, by lacking horns or tubercles on the head and pronotum, and by lacking a longitudinal furrow on the pronotum (Figs 26–28). With the anterior half of the body flatter than the posterior half and the flattened pronotum lacking a longitudinal furrow, *Platyphileurus* has an unusual appearance for a phileurine species. Its systematic position needs to be re-examined.

Here we describe the larva and pupa of *Platyphileurus felscheanus* repeatedly collected in bromeliad rosettes and reared to imagines, present data on their life history, and give new records on the occurrence of this bromelicolous species. We also explore whether characters of the immatures provide indications of the tribal placement of the genus.

### Beetles and bromeliads

Among the numerous insects recorded in bromeliad phytotelmata, beetles are typically represented by taxa with aquatic and semiaquatic habits. Larvae of Scirtidae are commonly found in bromeliad tanks (Picado 1913; Kitching 2000; Frank and Lounibos 2008; authors’ pers. obs.). Both, larvae and imagines of dytiscids in the genera *Desmopachria* Babington, 1841 and *Copelatus* Erichson, 1832 (formerly *Aglymbus* Sharp, 1880) occur in the water-filled leaf axils and prey on other insects there (Kitching 2000; Frank and Lounibos 2008; authors’ pers. obs.). In addition, species of *Phaenonotum* Sharp, 1882, *Lachnodacnum* Orchymont, 1937 and *Omicrus* Sharp, 1879 (Hydrophilidae) live in bromeliad phytotelms (Hansen and Richardson 1998; Kitching 2000; Frank and Lounibos 2008; authors’ pers. obs.).

Some few bromeliad associated species are Scarabaeidae (Champion 1913; Lüderwaldt 1915; Pereira et al. 1960; Huijbregts 1984; Costa et al. 1988; Cook 1998; Krell et al. 2002; Cave 2005; Howden 2010; F.Z. Vaz-de-Mello pers. comm. 2010; authors’ pers. obs.). Imagines of *Genuchinus* Westwood, 1874 (Cetoniinae) are frequently caught in bromeliads in Guatemala and Brazil (Howden 2010; Vaz-de-Mello pers. comm. 2010), but their life history is unknown. Imagines of a few species of *Bdelyrus* Harold, 1869 (Scarabaeinae) have been collected in bromeliads and seem to be closely associated with the plants (Pereira et al. 1960; Huijbregts 1984; Cook 1998; F.F. Albertoni pers. obs.). Larvae of *Desicasta laevicostata* (van de Poll, 1886) (= *Desicasta reichei* (Thomson, 1860); Cetoniinae) were found in living stalks of the epiphytic bromeliad *Vriesea sanguinolenta* Gogn. and Marchal in Panama and were reported eating the stalk tissue (Krell et al. 2002). One larva of *Trigonopeltastes delta* Forster, 1771 was collected two meters above the ground in the terrarium of *Tillandsia utriculata* L. in Florida. Even though, the larvae of *Trigonopeltastes delta* are known to develop in wood and other plant debris (Cave 2005).

## Material and methods

### Study areas

Bromeliads were collected from five rain forest areas in Santa Catarina state, southern Brazil, specifically on Santa Catarina Island (municipality of Florianópolis). Two sites are secondary forest areas: 1) Unidade de Conservação Ambiental Desterro (UCAD) (27°31'52"S, 48°30'45"W), a 491 ha forest reserve of the Universidade Federal de Santa Catarina (Zillikens and Steiner 2004), and 2) Santo Antônio de Lisboa (27°30'S, 48°31'W) about 2 km away from the former site (Zillikens et al. 2001). Three sites are sand dune habitats (“restinga”) with shrubby vegetation at 3) Campeche beach (27°40'38"S, 48°28'48"W), or with trees (“restinga arbórea”) at 4) Santinho-Moçambique beach (27°28'5"S, 48°23'13"W, 20 m a.s.l.) and 5) Pantano do Sul beach (27°46'52"S, 48°31'11"W, 7 m a.s.l.).

### Collection and rearing of beetles

To sample the associated animals we collected 412 bromeliads of six species from March 2002 to March 2006: *Nidularium innocentii* Lem. (n=99), *Aechmea lindenii* (E. Morren) Baker (n=141), *Aechmea nudicaulis* Griseb. (n=61), *Canistrum lindenii* (Regel) Mez. (n=60), *Vriesea vagans* (L. B. Sm.) L. B. Sm. (n=39), and *Hohenbergia augusta* Mez. (n=12). Whole plants were cut off at the base and examined leaf by leaf in the laboratory (Zillikens et al. 2005). The immature beetles were fixed in 70% ethanol, in Kahle’s solution, or in boiling water, and in the last case preserved in 80% ethanol; some larvae were kept alive to be reared.

All large beetle larvae and pupae found in the bromeliads were identified as scarabaeids. We prepared small rosettes of *Nidularium innocentii* from the innermost part of the plant (about 8–10 young leaves), washed with tap water to remove spiders and other predatory arthropods, or arranged freshly cut, clean bromeliad leaves to an artificial rosette in a funnel. Larvae and pupae were placed in the middle of the rosette and covered with 1–2 table spoons of humic leaf litter. The arrangement was covered with gauze and kept moist in the laboratory. Larvae were inspected about every second week to check their vitality and to replace the eaten up rosettes or leaves with new ones. The identification of *Platyphileurus felscheanus* was based on imagines obtained from these rearings.

In order to collect more specimens of *Platyphileurus felscheanus* and to learn about its life history, five additional bromeliads of the genera *Aechmea* Ruiz & Pav., *Nidularium* Lem. and *Vriesea* Beer. were collected between 2008 and 2012 in Florianópolis. In the field, we searched bromeliads for scarabaeid larvae. When a larva was present (n=6), the bromeliad was taken to the laboratory where it was kept upright in a plastic bucket. During the first days or weeks after collection the larvae were left in the bromeliads to observe their behavior. Thereafter, some bromeliad leaves were tied together in small rosettes and each larva was placed in the middle of this artificial rosette which were maintained in plastic pots (n=5). One larva was maintained in the original bromeliad until pupation. They were checked one to three times per week.

### Phylogenetic reasoning

A cladistics analysis at tribal or generic level is beyond the scope of this paper. In full consideration that the current tribal classification rests on entirely typological foundations, we apply consistently phylogenetic reasoning sensu Hennig (1982) and Watrous and Wheeler (1981) to interpret character states and their distribution to determine a possible placement of *Platyphileurus* in a current tribe.

### Material examined

***Platyphileurus felscheanus* Ohaus, 1910**

**The larval description is based on four third instar larvae with the following data: BRAZIL, Santa Catarina:** UCAD, Florianópolis city, in *Canistrum lindenii*, 18.ii.2002, J. Steiner leg. (DMNS ZE.15759); dto., in *Hohenbergia augusta* (plant no. 17), 15.iv.2002, A. Zillikens leg. (DMNS ZE.15761); dto., in *Aechmea lindenii* (plant no. 329), 27.iv.2004, A. Zillikens leg. (DMNS ZE.15760); Santo Antônio de Lisboa, Florianópolis, in *Aechmea nudicaulis* (plant no. 318), 24.iii.2004, A. Zillikens leg. (DMNS ZE.15758).

**Figures 1–4.**
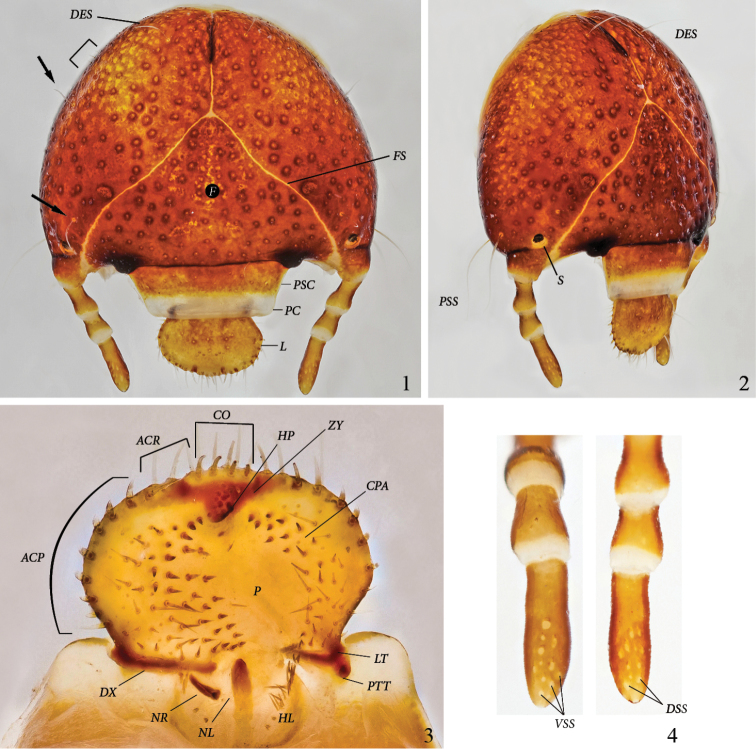
*Platyphileurus felscheanus* Ohaus, 1910, third instar DMNS ZE.15758: **1** head capsule frontal, arrows indicate setae, half rectangle indicates three minute setae, *DES – dorsal epicranial setae, F – frons, FS – frontal suture, L – labrum, PC – preclypeus, PSC – postclypeus* **2** head capsule fronto-lateral view, *DES – dorsal epicranial setae, PSS – post stemmatal setae, S – stemmata* **3** epipharynx, *ACP – acanthoparia, ACR – acroparia, CO – corypha, CPA – chaetoparia, DX - dexiotorma, HL – haptolochus, HP – haptomeral process, LT – laeotorma, NL – left nesium*, *NR – right nesium, P – pedium, PTT – pternotorma, ZY – zygum* **4** antennae, ventral and dorsal view, respectively, *VSS – ventral sensory spot*, *DSS – dorsal sensory spot*. Photos: C Grinter.

**Further larval material from which additional measurements were taken: BRAZIL: São Paulo:** 1 third instar (MZSP 010.247): Salesópolis, Estação Biológica de Boracéia, Atlantic rain forest, 23°32'S, 45°51'W, 4–12.ix.2008, S.A. Casari and M. Duarte (MZC-016-Entomologia de Campo) leg. [NEW STATE RECORD]. **Santa Catarina:** 1 larva fixed (MZSP 010.246): Florianópolis city, Santinho, restinga, in *Vriesea* cf. *friburgensis*, 27°28'42.4"S, 48°23'6.8"W, 2.iii.2008, A.G. Martins and F.F. Albertoni leg. (Fig. 1); 1 larva fixed (MZSP 010.245): Florianópolis city, Santo Antônio de Lisboa, in *Aechmea lindenii* (plant NA20), 4.ii.2004, A.F. Cordeiro and M. Manfredini leg.; 3 larvae (MZSP 010.248): same locality and plant species (plant NA19), 2.iii.2004, A.F. Cordeiro and M. Manfredini leg.; 1 larva (MZSP 010.249): same locality and plant species (plant NA44), 5.iv.2004, A.F. Cordeiro and M. Manfredini leg.; 1 larva (MZSP 010.250): same locality and plant species (plant no. 362), 15.ix.2004, A. Zillikens and J. Steiner leg.; 1 larva (MZSP 010.251): Florianópolis city, UCAD, Atlantic rain forest, in *Canistrum lindenii* (plant no. 74), 16.v.2003, A. Zillikens and J. Steiner leg.

**The pupal description is based on two pupae with the following data: BRAZIL: Santa Catarina:** 1 female pupa (reared from larva) (MZSP 010.252) Florianópolis city, Pantano do Sul, “restinga arbórea” in *Vriesea friburgensis* Mez., 06.ii.2008, A.G. Martins and F.F. Albertoni leg. (illustrated and photographed); 1 male pupa (reared from larva) (MZSP 010.253): Florianópolis, Santo Antônio de Lisboa, in *Aechmea* sp., 23.iii.2011, A.G. Martins and F.F. Albertoni leg. (photographed).

**Imagines of *Platyphileurus felscheanus* preserved: BRAZIL: Santa Catarina:** 1 male and 1 female (reared from larvae) (LANUFSC): Campeche, Florianópolis, restinga, *Aechmea nudicaulis* (plant no. 235 and 236), 28.xi.2003, A.F. Cordeiro leg.; 1 female (reared from larva) (DMNS ZE.20187): UCAD, Florianópolis, in *Aechmea lindenii* on rock (plant no. 63), 14.x.2002 (emergence: 29.x.2002), A. Zillikens leg.; 1 male (reared from larva) (MZSP): Florianópolis, Santo Antônio de Lisboa, in *Aechmea caudata* Lindm., 26.iv.2008, A.G. Martins and F.F. Albertoni leg.; 1 female (reared from larva) (DMNS ZE.20188), same locality, in *Aechmea lindenii*, 5.vii.2004 (emergence: 8.x.2004), J. Steiner and A. Zillikens leg.; 1 female (reared from larva) (LANUFSC): same locality, in *Aechmea* sp., 23.iii.2011 (emerged: 09.xi.2011), A.G. Martins and F.F. Albertoni leg.; 1 male (LANUFSC): same locality, among the litter of *Hohenbergia augusta*, 27.xi.2012, F.F. Albertoni and J. Linemburg Jr. leg.

**Additional material:** Two last larval instar exuvia and one pupal exuvia (MZSP) of the reared *Platyphileurus felscheanus* larvae.

To assess the differences of apical setal patterns of pupae between different species of Dynastinae, pupae of the following species of Phileurini and Oryctini from the immature collection of MZSP were studied (for descriptions see Vanin et al. (1983), Costa et al. (1988)):

***Homophileurus luederwaldti* (Ohaus, 1910**)

**BRAZIL: São Paulo:** One pupa reared from larva (genital ampulla damaged, sex not determined) (MZSP) Itanhaém, 12.i.1978, L.R. Fontes leg. in nest of *Microcerotermes* sp. (Isoptera).

***Trioplus cylindricus* (Mannerheim, 1829**)

**BRAZIL: São Paulo:** One male pupa reared from larva (MZSP), São Paulo city, Cidade Universitária (USP), 09.i.1979, S.A. Vanin & C. Costa leg. in decaying tree trunk.

***Strategus validus* Fabricius, 1775**

**BRAZIL: São Paulo:** One male pupa reared from larva (MZSP), Peruíbe city, 25–27.v.1982, exp. MZUSP leg.

***Mystacella* sp. (Diptera: Tachinidae: Exoristinae: Goniini**).

**BRAZIL, Santa Catarina:** 2 imagines (reared from pupae), 2 puparia and 1 puparial exuvia of *Mystacella* sp. (MZSP) from 1 *Platyphileurus felscheanus* larva reared to pupa (MZSP): Florianópolis city, Pantano do Sul, “restinga arbórea”, in *Vriesea friburgensis*, 02.ii.2008, A.G. Martins and F.F. Albertoni leg.

### Repositories

DMNS: Denver Museum of Nature & Science (Denver, CO, U.S.A.); MZSP: Museu de Zoologia da Universidade de São Paulo (São Paulo, Brazil); LANUFSC: Laboratório de Abelhas Nativas da Universidade Federal de Santa Catarina (Florianópolis, SC, Brazil).

## Results

### Description of the third instar larva of *Platyphileurus felscheanus* Ohaus, 1910

Figs 1–13

Terminology after Böving (1936), Morón (1986) and Ratcliffe and Skelley (2011).

**Body length:** 34–62 mm (*x*=46 mm; SD=12.5 mm; n=7 preserved specimens); dehydrated or otherwise contracted specimens measured as low as 26 mm.

**Cranium** (Figs 1–2): Width of head capsule: 4.9–5.5 mm (*x*=5.2 mm; SD=0.2 mm; n=12). Reddish brown, strongly and moderately densely punctate. Light yellow sharp frontal suture (*FS*) reaches antennal base. Stemma (*S*) present at antennal base, close to frontal suture. Anterior angles of epicranium with 2 long, thin setae behind distal side of antennal base (*PSS*) (1 on dorsal, 1 on ventral side of epicranium). Up to 4 microscopic setae basoventrally of *DES* (often missing). A long, thin seta behind stemma close to frontal suture (arrow); 1 lateral, long, thin seta just behind middle of epicranium, and 1 lateral seta close to epicranial suture (arrow). Frons without setae.

**Clypeus** (Figs 1–2): Trapezoidal with straight sides. Postclypeus (*PSC*) orange brown, with punctures smaller and sparser than on cranium. One external seta on each side. Preclypeus(*PC*) white, without punctures.

**Labrum** (*L*) (Figs 1–2): Orange brown with lighter anterior margin. Broadly oval, with rounded, not angulate, lateral margins; with several discal points similar to those of postclypeus(*PSC*); without posterior labral setae, but with 1 or 2 lateral setae on each side, 1 in front of labral base and 1 close to anterior margin. Anterior margin slightly trilobate, with one seta each on shallow outer lobes and 2 setae on the stronger middle lobe.

**Epipharynx** (Fig. 3): Form transversely suboval, asymmetrical, with left lateral margin obtusely angulate in the middle. Right and left chaetoparia (*CPA*) with 53–60 and 33–48 setae, respectively; up to 10 sensilla among setae on each side. Acroparia (*ACR*) with 3 to 4 thick setae each. Left Acanthoparia (*ACP*) with 5 to 9 thick setae, anterior ones thicker and longer. Right acanthoparia with 6 to 9 thick setae, anterior ones thicker and longer. Pedium (*P*) extended to the left. Corypha (*CO*) with 10 thick setae. Zygum (*ZY*) brown, triangular, with ventral angle forming a blunt haptomeral process(*HP*). Laeotorma (*LT*) shorter than dexiotorma(*DX*). Pternotorma (*PTT*) blunt, rounded. Right nesium (*NR*) caudolaterally shifted and enlarged, forming a sharp, ventrally extending tooth. Left nesium (*NL*) caudally elongated with sense cone on anterior tip. Haptolachus (*HL*) without setae except left margin bearing about 20 thin, long setae. Crepis missing.

**Figures 5–8.**
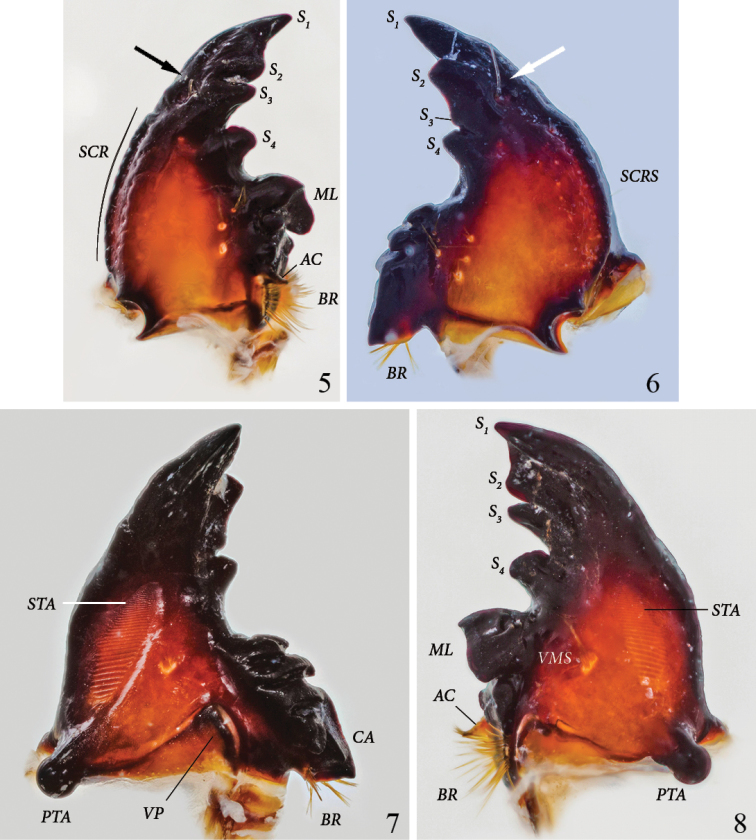
*Platyphileurus felscheanus* Ohaus, 1910, third instar mouth parts DMNS ZE.15758: **5** left mandible dorsal view, *arrow indicates setae, AC – acia, BR – brustia, ML – molar lobe, S_1-4_ – scissorial teeth, SCR – scrobis* **6** right mandible dorsal view, *arrow indicates setae, BR – brustia, S_1-4_ – scissorial teeth, SCRS – scrobis seta* **7** right mandible ventral view, *BR – brustia, CA – calx, PTA – postartis, STA – stridulatory area, VP – ventral process* **8** left mandible ventral view, *AC – acia, BR – brustia, ML – molar lobe, PTA – postartis, S_1-4_ – scissorial teeth, STA – stridulatory area, VMS – ventral molar setae*. Photos: C Grinter.

**Left mandible** (Figs 5, 8): Form falcate. Scissorial area with S_1_ and S_2_ distant but bridged by flat area forming broad apical blade, separated from S_3_ by acute scissorial notch. S_4_ of similar size as S_3_, blunt, of cylindrical appearance in ventral view, separated from S_3_ by acute and deep notch. Mandible dorsally with 1 long discal seta in front of labium at level of S_3_ (arrow). Outer margin convex. Scrobis (*SCR*) with 1 short, thin seta. Dorsal area adjacent to scrobis with 2 rows of 7 sensorial pits. Dorsal area adjacent to molar crown with 3 setae. Acia (*AC*) well developed, with brush of apical setae. Brustia (*BR*) with 12 long setae. Ventral surface with elongate-oval stridulatory area (*STA*) with about 30 narrowly separated, subparallel ridges. Molar area with a tuft of 8 ventral molar setae(*VMS*) (setae very close together and difficult to count). Molar lobe (*ML*) large, forming a dorsoventral ridge, not subdivided. Molar crown with 2 lobes. Postartis (*PTA*) large, spherical. Ventral process triangular with rounded tip.

**Right mandible** (Figs 6–7): Form falcate. Scissorial area with S_2_ separated from S_1_ by obtusely angled notch. S_2_ and S_3_ distant but bridged by flat area, S_3_ hardly developed as a denticle but with deep, acute notch separating it from S_4_. S_4_ triangular, as elevated as S_2_. Mandible with 1 long discal seta in front of labium at level of S_3_ (might be lacking or broken off) (Fig. 6, arrow). Outer margin convex. Scrobis with 1 short, thin seta(*SCRS*). Dorsal area of scrobis with an inner row of 5 and an outer row of 9 sensorial pits. Dorsal area adjacent to molar crown with a row of 4 white pits with 0, 4, 1, and 2 setae, respectively; discally with longitudinal row of 3 distinct, white pits with 3, 1, and 2 setae, respectively. Ventral surface with elongate oval, anteriorly tapering, stridulatory area (*STA*) with about 30, narrowly separated, subparallel ridges. Molar area with 5 ventral molar setae. Molar crown with 3 blunt ridges. Calx (*CA*) ventrally and dorsally ending in slightly blunt denticle. Brustia (*BR*) with about 15 setae. Postartis (*PTA*) large, spherical. Ventral process (*VP*) suboval, elongated laterally.

**Figures 9–10.**
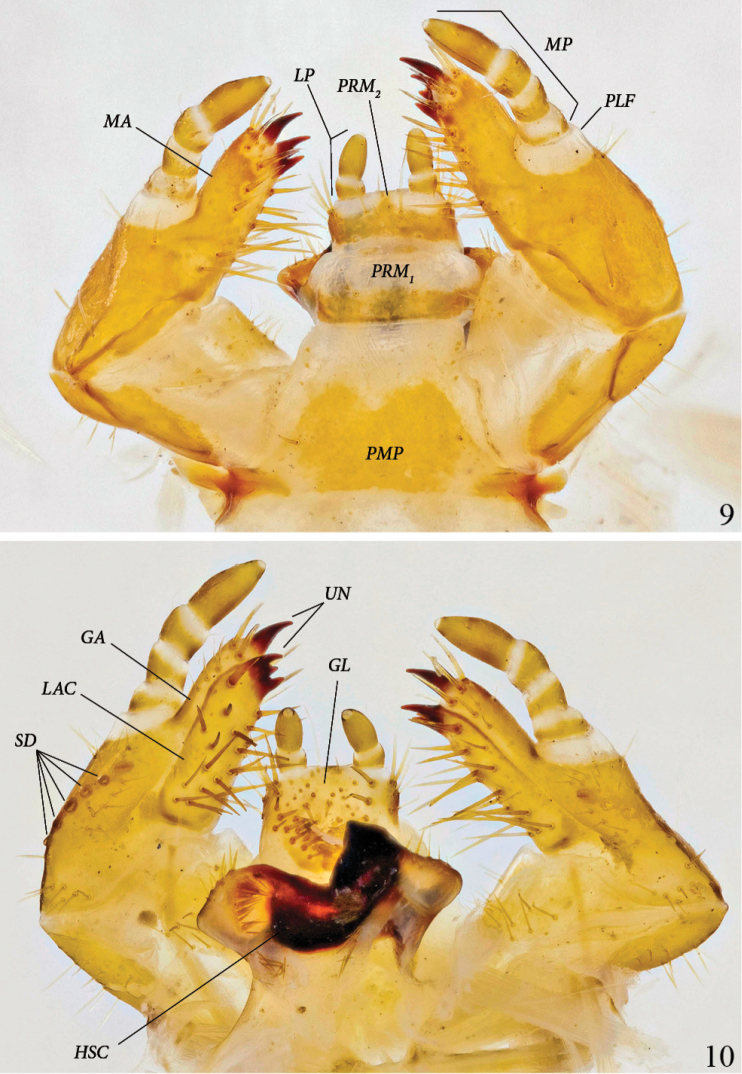
*Platyphileurus felscheanus* Ohaus, 1910, third instar mouth parts DMNS ZE.15758: **9** maxilla and labium ventral view, *LP – labial palpus, MA – mala, MP – maxillary palpus, PLF – palpifer, PMP – post-mentum, PRM_1, 2_ – Prementum 1 and 2* **10** maxilla and labium dorsal view, *GA – galea, GL – glossa, LAC – lacinia, HSC – hypopharyngeal sclerome, SD – stridulatory teeth, UN – uncus*. Photos: C Grinter.

**Maxilla and labium, ventral view** (Fig. 9): Galea and lacinia fused, forming mala(*MA*). Ventral inner margin and apical area of mala with 7–8 strong, long setae, and another 5 or more on inner side of mala. Maxillary palpus (*MP*) 4-segmented; palpifer (*PLF*) white, membranous; spindle-shaped apical segment about twice as long as each preceding segment. Third segment with 2 strong, ventral setae. Mentum subdivided into 3 segments: yellow post-mentum (*PMP*) with one basolateral and one apicolateral seta on each side, white prementum 1 (*PRM_1_*) with orange base and 2 discal setae, orange prementum 2 (*PRM_2_*) with 2 setae on white base of each palpus. Labial palpus (*LP*) 2-segmented, spindle-shaped apical segment twice as long as basal segment.

**Maxilla and labium, dorsal view** (Fig. 10): Galea and lacinia not fused. Galea (*GA*) with one apical uncus (*UN*); apicodorsal margin of galea with 3–4 setae. Lacinia (*LAC*) with 3 touching subterminal unci; dorsal surface of lacinia with 17–19 long, strong setae. Stridulatory area with 1 large, darker stridulatory tooth and a row of 4 to 6 smaller, rounded, sometimes more lightly colored teeth(*SD*), reaching longitudinally towards base of stipes. Glossa (*GL*) with 23–31 long and 12–13 very short setae. Hypopharyngeal sclerome (*HSC*) asymmetrical, with median rectangular incision, right side with one triangular, tooth-like process produced dorsally; left side neither elevated nor protruded. Left lateral lobe with 7 setae on margin, in middle with dense, longitudinal row of about 15 broad setae directed mesally and a few more basally; right lateral lobe with 8 setae on anterior margin and an oblique row of 6 setae basally at caudomedian border of hypopharyngeal sclerome.

**Antenna** (Fig. 4): Four-segmented with fourth antennomere the longest, about 1.7 times as long as third, second antennomere slightly shorter than fourth, first antennomere slightly shorter than third but thicker than others. Terminal antennomere with 12–13 ventral sensory spots (*VSS*) and 13–15 dorsal sensory spots(*DSS*); apex with 1 sensory spot.

**Figures 11–13.**
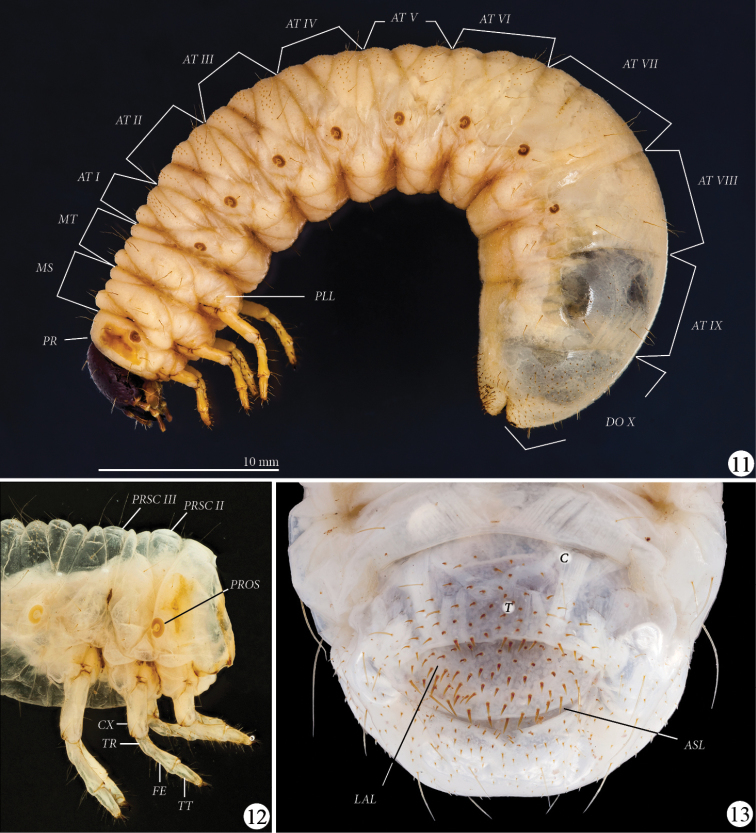
*Platyphileurus felscheanus* Ohaus, 1910, third instar. **11** lateral view MZSP 010.245, *AT I-IX – abdominal tergites I to IX, DO X – dorsum X, MS – mesothorax, MT – metathorax, PLL – pleural lobes, PR - prothorax* **12** thorax and legs lateral view DMNS ZE.15758, *CX – coxa, FE – femur, PROS – prothoracic spiracle, PRSC II – prescutum II, PRSC III – prescutum III,, TR – trochanter, TT – tibiotarsus* **13** larval raster DMNS ZE.15760, *ASL – anal slit, C – campus, LAL – lower anal lip, T – teges*. Photos: FF Albertoni (**11**), C Grinter (**12–13**).

**Thorax** (Figs 11–12): Prothoracic spiracle (*PROS*) 0.48–0.54 mm wide, 0.76–0.80 mm long. Respiratory plate light brown, ovally C-shaped, with ends touching. Bulla barely prominent. Respiratory plate with about 35 holes across diameter at middle. Dorsum of pronotum (*PR*) and prescutum II (*PRSC II*) and III (*PRSC III*) each with 2 lateral, long, slender setae, otherwise glabrous.

**Legs** (Figs 11–12): Tarsal claws falcate, all similarly curved and similar in size, with 1 basal seta and 1 seta in the middle of inner side. Tibiotarsus (*TT*) with 4 apical setae on outer side of the base of claw, and with 2 circular rows of 6 long setae. Femur (*FE*) with 2 circular rows of 4–5 long setae and accessory setae. Trochanter (*TR*) with 5–6 ventral setae. Coxae (*CX*) with 4 setae. Setae of legs light brown to transparent, thin.

**Abdomen** (Fig. 11): Spiracles of similar size as prothoracic spiracle; last one smaller; rounder than prothoracic spiracle. One long seta on stigma area behind each abdominal spiracle II to VII, no seta behind spiracles I and VIII. Pleural lobes (*PLL*) with 2 long setae. Pedal area with 1 central and 2 lateral long setae per segment. Abdominal tergites (*AT*) I–VII with many tiny, short, dark, spike-like setae, not arranged in rows. Abdominal tergite IX laterally with dark, sparse minute, spike-like setae. Dorsum X (*DO X*) completely covered with such setae.

**Raster** (Fig. 13): Surface without palidia. Campus (*C*) with 6 slender, moderately long setae. Teges (*T*) with about 110–115 shorter, thorn-like setae (some longer) anterior of transverse anal slit(*ASL*), slightly bent toward anal slit. Lower anal lip (*LAL*) with about 80–100 thinner, shorter, minute, thorn-like setae, some thin and long. Setae not arranged in any pattern.

### Description of the pupa of *Platyphileurus felscheanus* Ohaus, 1910

Figs 14–25, 34

**Female pupa** (Figs 14–16, 18, 20, 22, 24–25, 34):

Length 23.9 mm; largest width 10.4 mm.

Adecticous; exarate; body oblong, smooth, apparently glabrous but with microsetae covering whole body (best seen at magnification > 50 ×), apex of tergite IX with dense tuft of setae seen in dorsal and ventral views; abdominal segments constitute almost two thirds of whole body; yellowish-brown before and after fixation, gin-traps and spiracular rings darker and more strongly sclerotized.

**Head:** epistomal suture incomplete at middle, clypeus with shallow depression, clypeolabral suture slightly marked.

**Figures 14–18.**
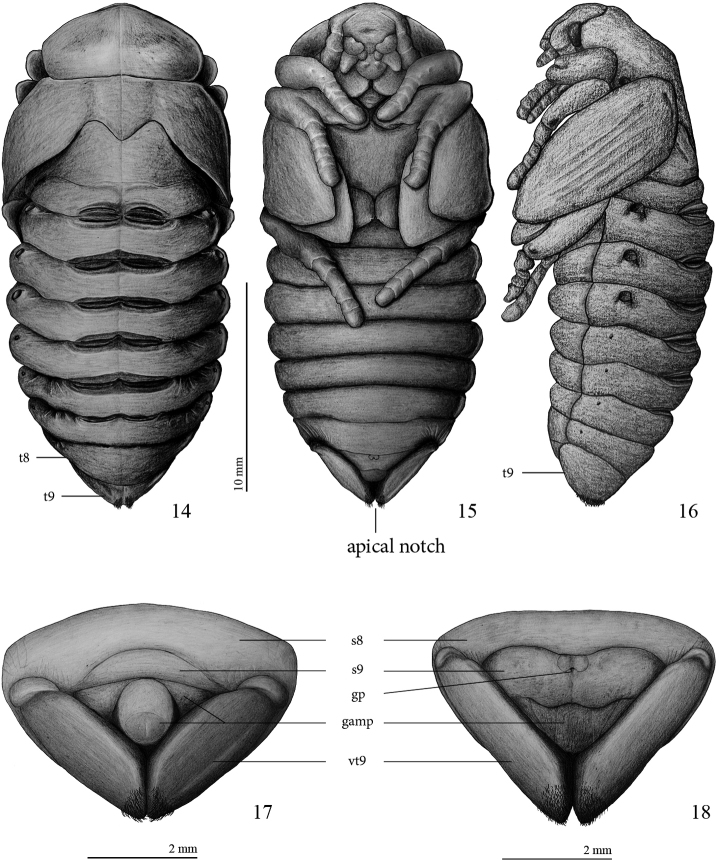
*Platyphileurus felscheanus* Ohaus, 1910, pupa: **14** female pupa dorsal view, *t8, t9 – tergite 8 and 9*, respectively **15** female pupa ventral view **16** female pupa lateral view **17** male pupa, ventral view of apex with genital ampulla **18** female pupa, ventral view of apex with genital ampulla. Legends: *s8, s9 – sternite 8 and 9, respectively; gamp – genital ampulla; gp – genital pore, vt9 – ventralized tergite 9*. Drawings: FF Albertoni.

**Pronotum** (Fig. 14): almost twice as wide as long; pentagonal, widest at middle, narrowed anteriorly, lateral margins rounded, medially with slightly transversal groove. Scutellum pentagonal, 1.2 times longer than wide.

**Pterotheca** (Figs 15–16): close to body, curved ventrally, extending posteriorly to second abdominal segment.

**Abdomen:** segments 1–7 widened transversally, about 6 times wider than long; segments 3, 4 and 5 widest; segment 8 longer on dorsal view than ventrally, about 4.5 times wider than long (Fig. 14); tergite 9 (*t9*) triangular, laterally extended to ventral side (see Figs 17–18) and with apical notch in ventral view; each side of apical notch covered with a tuft of yellowish, short, thin setae that do not extend ventrally, laterally, or dorsally (Figs 14–16, 18, 20, 22, 24–25); sternite 9 with genital pore; 4 pairs of functional spiracles, first pair almost completely concealed by pterothecae, other 3 pairs protruding about 0.2 mm (Figs 14, 16), 4 pairs of vestigial spiracles on laterotergites 5–8 (Fig. 16). Six pairs of gin-traps mid-dorsally in intersegmental region, the largest and most sclerotized between segments 1 and 2, those between segments 2 and 3, 3 and 4, 4 and 5, and 5 and 6 of same size, those between segments 6 and 7 smallest.

**Figures 19–25.**
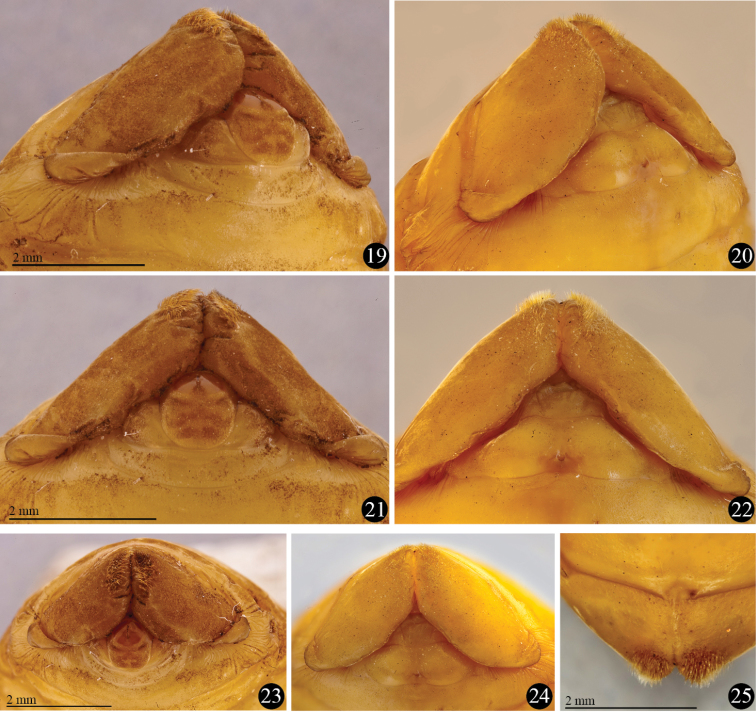
*Platyphileurus felscheanus* Ohaus, 1910, pupal apex showing genital ampulla, setose apex of ninth tergite and base of ventralized tergite 9: **19** male, ventro-lateral view **20** female, ventro-lateral view **21** male, ventral view **22** female, ventral view **23** male, frontal view **24** female, frontal view **25** female, dorsal view. Photos: FF Albertoni.

**Female genital ampulla** (*gamp*) (Figs 18, 20, 22, 24): at middle with 2 almost parallel lines slightly convergent anteriorly and 2 diagonal lines, 1 per side.

**Male pupa** (Figs 17, 19, 21, 23):

Length: 19.0 mm; largest width 10.1 mm.

Most characteristics as in female but basal part of ventral side of ninth tergite (*vt9*) with much stronger constrictions than in female (Figs 17–25). In males, they resemble 2 lateral lobes, whereas in females they are less folded (Figs 19–24). Sternite 9 (*s9*) anteriorly convex in males, in females with different form (see Figs 17–22).

**Male genital ampulla** (*gamp*) (Figs 17, 19, 21, 23) divided into 2 parts: a rounded structure and a narrow, trapezoidal band strongly emarginated at middle, surrounding partially the rounded structure; rounded structure with circular, glabrous area at apex, glabrous area with longitudinal, dark line impressed at apex.

### Life history

In 14 ground-growing bromeliads, we collected 19 larvae of *Platyphileurus felscheanus*, 13 of them in *Aechmea lindenii*, three in *Aechmea nudicaulis*, one in *Canistrum lindenii*, one in *Hohenbergia augusta*, and one in *Nidularium innocentii*. The highest number of larvae in one bromeliad was four in a single *Aechmea lindenii*, but the majority of bromeliads had only one larva.

Second instars were found at the base of more external leaves of the rosette among leaf litter. No damage to bromeliad leaves near the larvae was observed. Third instars were always found in the center of the rosette with the surrounding leaves strongly damaged (Figs 32–33).

Coincidentally, when the bromeliad leaves started to dry and consequently to decompose, the larvae stopped feeding and started pupation. They built their pupal chamber in the center of the leaf rosettes or among leaves near the center, using leaf litter, twigs, and humus that had accumulated there. The larval exuvia was pushed to the rear of the pupal chamber (Fig. 34). Observations of the second phase are summarized in Table 1.

**Table 1.** Results of second phase of study on *Platyphileurus felscheanus* life history.

	Pantano do Sul, restinga arborea		Santinho/Mocambique, restinga arborea	Sto Antonio		
**Bromeliad searched/ collected**	6 *Vriesea friburgensis* (Fig. 31) No damage to plant		3 *Aechmea* spp. Plant senescent	12 *Aechmea caudata* Plant apparently not injured.	5 *Aechmea* sp. and 3 *Nidularium* sp. Plant apparently not injured.	
**Larvae found**	1 second instar larva (n° 1); 2.iv.2008. In outer leaf axils with much litter, but moved into the centre of rosette. Smaller than specimens n° 2 and 3.	1 second instar larva (n° 2); 2.iv.2008. Outer leaf axils among leaf litter, but moved into the centre of rosette. Smaller than n° 3.	1 second instar larva (n° 3); 2.iii.2008. Between dry external leaves, later moved slowly into the central rosette. Moulted on 7.iii.2008.	1 third instar larva (n° 4) (Fig. 32) in *Aechmea caudata* 26.iv.2008. Down in center of rosette.	1 third instar larva (n° 5) in *Aechmea* sp. 23.iii.2011. Down in center of rosette.	1 third instar larva (n° 6) in *Aechmea* sp. Just next to the bromeliad of n° 5; Down in center of rosette.
**Ate what**?	Ate basal parts of leaves.	Ate white basal part of leaves.	Ate dry leaves; later basal part of green leaves and stalk.	Ate the basal parts of leaves.	Ate the basal (and green) parts of leaves.	Ate the basal (and green) parts of leaves.
**Pupa**	6.ii.2009 starts pupal chamber, 10.ii pre-pupa, 15.ii. pupa, (fixed).	25.xii.2008 pupa observed being parasitized by Tachinidae (Diptera) (Figs 36–37). (4 puparia of *Mystacella* sp.) (Fig. 35).	22.ix.2008 larva fixed.	Before 15.ix.2008	Before 04.x.2011. Fixed on 15.x.2011.	On 08.x.2011.
**Remarks**	Female pupa.	9.i.2009 emergence of 2 flies, fixed together with puparia.	Larva fixed with boiling water, preserved in ethanol 90%.	Male imago emerged 5.x.2008. Gnawed wood. Stayed most part of the time buried. Died 24.xii.2008.	Male pupa. It seemed to pupate before final growing time and turns to smaller pupa than n° 1.	pupa smaller than n°. 1 and 5 Female imago emerged on 10.xi.2011. Ate banana. Stayed most part of the time buried. Died on 9.xii.2011.

Parasitism by Tachinidae flies was observed in one pupa (Fig. 35). As the bromeliad with the larva was kept open in the laboratory, we do not know whether parasitism occurred in the laboratory or in the field. The chamber of the affected pupa seemed crudely done, mainly built with plant fiber and missing a more consistent humus wall.

## Discussion

### Pupal morphology

Generally, pupae of Scarabaeidae are of uniform appearance, mainly differing by adult characters such as horns. Usually they do not have pupa-specific ornamentations or setae for chaetotaxy analysis unlike pupae of other beetle families which might be the reason for the poor attention that scarabaeoid pupae have gained in the past (see Costa et al. 1988; Costa and Ide 2008).

Several descriptions of pupae from Dynastinae tribes, such as Cyclocephalini, Phileurini, Oryctini point out the thin golden setae present at the apex of 9^th^ abdominal segment but rarely mention the shape of the 9^th^ tergite and the genital ampulla (Phileurini: Vanin et al. 1983; Morelli [1991]; Neita-Moreno and Ratcliffe 2010, 2011; Cyclocephalini: Neita-Moreno et al. 2007; Neita-Moreno and Morón 2008; Oryctini: Ratcliffe and Chalumeau 1980; Costa et al. 1988; Morelli 1997; Alvarez Castillo et al. 1998; Neita-Moreno and Orozco 2009; Pardo-Locarno et al. 2009), characters which seem to vary within Scarabaeidae. Our examination of pupae from Phileurini and Oryctini revealed that they can be distinguished from that of *Platyphileurus felscheanus* based on the form of the 9th tergite and genital ampulla and the distribution and size composition of those apical setae.

### Comparison with other scarabaeid pupae

In a pupa of *Homophileurus luederwaldti* examined (length: 25.3 mm, largest width: 13.0 mm; Phileurini) the setation on the apex differs from that of *Platyphileurus felscheanus* by covering a much wider area, until the middle of the ventral part of *t9*, and the setae also extend slightly towards the dorsal side. The apical notch is more open than it is in *Platyphileurus felscheanus*. The genital ampulla region was damaged, and could not be analysed. In a male pupa of *Trioplus cylindricus* (length: 22.0 mm, largest width: 9.3 mm; Phileurini), the setae on the apical tergite are similar to the pupa of *Homophileurus luederwaldti*. They cover a larger area, spreading laterally towards the dorsal region, but almost do not change in density and sizes as in *Homophileurus luederwaldti*. The rounded structure of the genital ampulla is wider posteriorly and narrowed from middle to base, thus not rounded or elliptical. There are two longitudinal marks, one smaller anteriorly and the longer posteriorly. In a male pupa of *Strategus validus* (length: 60 mm, largest width: 27 mm; Oryctini), the rounded structure of the genital ampulla is elliptical with one longitudinal mark at the apex and thus, similar to that of *Platyphileurus felscheanus*. Tergite 9 has a transversal depression laterally (not present in *Platyphileurus felscheanus*); pubescence of uniform, small setae covering a wide area throughout the segment, ventrally covering almost the whole area extending laterally unto the edge of the lateral depression; and an apical notch connected mesally by V-shaped fold (absent in *Platyphileurus felscheanus*).

Furthermore, in *Hemiphileurus elbitae* Neita-Moreno and Ratcliffe 2010 (Phileurini), the apex of *t9* appears to be more densely setose over a larger area, and with longer setae (Neita-Moreno and Ratcliffe 2010) than in *Platyphileurus felscheanus*. The apices of male and female pupa of *Homophileurus tricuspis* Prell, 1914 (Phileurini) have setae that extend significantly more sidewards (Neita-Moreno and Ratcliffe 2011) than in *Platyphileurus felscheanus*. The pupa of *Phileurus affinis* Burmeister, 1847 (Phileurini) has *t9* covered with small and abundant setae on the apex, but there is no detailed illustration to compare (Morelli [1991]). The female pupa of *Aspidolea singularis* Bates, 1888 (Cyclocephalini) has the setae on *t9* similarly distributed as those of *Platyphileurus felscheanus*, but dorsally and laterally the shape of *t9* is different. In the former, it is almost as long as wide, and in the latter it is two times wider than long. In addition *t8* is more sharpened medially and projected towards the apex, whereas it is rounded in *Platyphileurus felscheanus*. The pupa of *Ancognatha ustulata* Burmeister, 1847 (Cyclocephalini) has setae widely distributed ventrally on *t9* (Neita-Moreno and Morón 2008).

Even though the knowledge of pupae is much more limited than that of larvae, pupae do have some exclusive characters such as setae, spurs, tubercles and modified spiracles (Costa and Ide 2008). As demonstrated here, the apex of the abdomen, namely the shape of *t9*, genital ampulla, and setal pattern could be a source of important characters for distinguishing between subfamilies, tribes, and genera of Scarabaeidae and perhaps could even be used in systematic and phylogenetic analyses, but need to be much more comprehensively studied. Altogether, we consider the data from pupal morphology presented here to be too limited to contribute to the discussion of the systematic placement of *Platyphileurus felscheanus*.

### Life history

Dynastinae larvae frequently feed on decaying plant matter, especially wood (Ritcher 1958; Carlson 1991; Ratcliffe et al. 2002), although food sources vary between species and include roots of living plants, especially monocots, and general organic matter (Ritcher 1958; Gassen 1989). Lourenção et al. (1999) reported larvae of *Strategus* Hope, 1837 (Oryctini) as a pest of Arecaceae feeding on live plant tissue. Larvae of Oryctini and Cyclocephalini are often associated with feeding on live plant tissue, unlike those in the tribes Phileurini and Dynastini that are known to feed on decaying wood or humus associated with decaying vegetable matter. Thus *Platyphileurus felscheanus* would be the first known exception in the Phileurini tribe.

Krell et al. (2002) reported larvae of a species of Cetoniinae feeding on living bromeliad tissue and already referred to an unidentified large dynastine larva collected by Lüderwaldt (1915) in bromeliads at Colonia Hansa, Blumenau, in Santa Catarina state, Brazil. Nevertheless, this is the first report of identified larvae of Dynastinae feeding on living leaf tissue (Fig. 32) and the only species of Dynastinae known so far whose larvae develop in bromeliad rosettes.

Considering the place where we found the younger larvae, females most likely lay eggs into the external leaf axils that are rich in decaying plant matter and humus, and usually dryer than the water-filled central rosette. Our observations suggest that the larvae initially feed on dead decaying vegetal matter and then migrate into the center of the rosette to feed on the white basal part of living leaves. It is during this second phase that the larvae gain most weight.

The construction of pupal chambers in Dynastinae species is poorly documented. Larvae of *Phileurus hospes* Burmeister, 1847 built their pupal chamber only with humus from the decaying tree trunk in which the larvae were found (F. F. Albertoni pers. obs.). The termitophilous species *Homophileurus luederwaldti*, *Actinobolus tribolus* Luederwaldt, 1910 and *Actinobolus radians* Westwood, 1841 used their own faeces and soil from the termite nest to build their pupal chambers (Luederwaldt 1911). *Homophileurus tricuspis* Prell, 1914 seemed to make a hole in the termite nest wall where the larvae delimited their pupal chamber (Neita-Moreno and Ratcliffe 2011). *Trioplus cylindricus* larvae built their pupal chamber excavating the wood or using fragments of wood (Vanin et al. 1983). As is the case with *Platyphileurus felscheanus*, these larvae seem to have opportunist behaviour by using the substrate next to them. The location, size, and the material used by *Platyphileurus felscheanus* for building its pupal chamber (Fig. 34) resembles those of another bromeliad associated insect, the lepidopteran *Geyeria decussata* (Castniidae) (Albertoni et al. 2012) except for the fact that the beetle larva does not use silk.

According to Ritcher (1958), most Scarabaeidae push the last larval exuvia to the rear of pupal chamber during pupation, but in Rutelinae and most Dynastinae the last larval exuvia splits longitudinally along the middorsal line and the pupa stays inside the larval exuvia. However, in a few genera such as *Oryctes* Hellwig, 1798 and *Strategus* Hope, 1837 (both Oryctini) the larval exuvia is pushed to the rear of the pupal chamber as was observed with *Platyphileurus* (Fig. 34).

Among the South American Coleoptera that are hosts of Tachinidae, Chrysomelidae and Scarabaeidae are the families with the highest numbers of parasitized species (Guimarães 1977), but none of the 7 genera of Goniini listed in Guimarães’s catalogue parasitized Coleoptera. Neither was the monotypic genus *Platyphileurus* Ohaus, 1910 registered as a host, nor was *Mystacella* van der Wulp, 1890 (Figs 36–37) registered as parasite for any beetle species. In North America, *Mystacella* spp. was recorded as parasitizing Lepidoptera of the families Arctiidae and Noctuidae and *Mystacella chrysoprocta* (Weidemann, 1830) parasitizing Scarabaeidae of the genus *Xyloryctes* Hope, 1837 (Arnaud 1978). Thus, our observations constitute a new host-parasitoid association.

### Transfer of *Platyphileurus* from Phileurini to Oryctini on the basis of larval and adult characters

Since the species studied here was not only described as two species, *Platyphileurus felscheanus* and *Surutu jelineki*, but also as belonging to different tribes, namely Phileurini and Cyclocephalini, it is appropriate to explore whether larval characters can contribute to resolving its tribal classification.

**Antennal sensory spots:** Larvae of Cyclocephalini are characterized by 2, 3, or 4 sensory spots on the last antennomere plus an apical one (Ritcher 1944; Remedi de Gavotto 1964; Morelli and Alzugaray 1994; Ramírez-Salinas et al. 2004; Neita-Moreno et al. 2007; Bezerra de Souza et al. 2014). With 12 to 13 dorsal and 13 to 15 ventral sensorial spots on the apical antennomere, *Platyphileurus felscheanus* would be the only known exception in this tribe.

Likewise, the species would have the highest number of antennal sensilla ever recorded in Phileurini larvae. So far, *Homophileurus integer* (Burmeister, 1847) had the highest number with 5 dorsal and 8 ventral sensory spots (Ratcliffe and Skelley 2011); most Phileurini have a lower number. In Dynastinae, a similarly high number of antennal sensilla was only found in the Dynastini species *Xylotrupes gideon* s.l. (Linnaeus, 1767) (Bedford 1974) and *Chalcosoma atlas* (Linnaeus, 1758) (Bedford 1976), in *Oryctoderus* (Oryctoderini; Bedford 1974), and in several species of the tribe Oryctini, namely a few *Strategus* species (Ritcher 1944; Ratcliffe and Chalumeau 1980; Costa et al. 1988; Morón and Ratcliffe 1990), *Heterogomphus chevrolati* Burmeister, 1847 and *Enema endymion* (Chevrolat, 1843) (Ratcliffe 2003), *Coelosis biloba* (Linnaeus, 1767) (Pardo-Locarno et al. 2006), *Oryctes monoceros* (Olivier, 1789) (Marcuzzi et al. 1977), *Oryctes boas* Fabricius, 1775 (Oberholzer 1959), *Trichogomphus fairmairei* Arrow, 1919, *Scapanes australis* (Boisduval, 1835) (Bedford 1974), and *Podischnus agenor* (Olivier, 1789) (Pardo-Locarno et al. 2009). With outgroup taxa (Rutelinae, Melolonthinae) having only 1 to 4 antennal sensilla (Ritcher 1966), and a high number of sensilla as in *Platyphileurus* not occurring in other Dynastinae tribes, this character is likely to be an apomorphy of Dynastini, Oryctoderini, and Oryctini.

**Larval mandibles:** The shape of the larval left mandible of *Platyphileurus* resembles most the larval mandibles of some Oryctini species. Particularly the large, blunt scissorial denticle S_4_ is most commonly found in Oryctini, namely in *Strategus splendens* (Palisot de Beauvois, 1809) (Ritcher 1944); *Strategus jugurtha* (Burmeister, 1847) (smaller, but blunt; Morón and Ratcliffe 1990); *Podischnus agenor* (slightly incised; Pardo-Locarno et al. 2009; Neita-Moreno and Orozco 2009), *Heterogomphus chevrolati* and *Enema pan* Fabricius, 1775 (Ratcliffe 2003), *Heterogomphus dilaticollis* Burmeister, 1847 (Neita-Moreno and Orozco 2009), *Trichogomphus fairmairei*, *Scapanes australis* (Bedford 1974), and thinner, but still blunt in *Heterogomphus pauson* Perty, 1830 (Alvarez-Castillo et al. 1998). It is also found in Dynastini, namely in *Megasoma elephas* Fabricius, 1775 (Morón 1977). In Cyclocephalini, S_4_ is mostly missing, only being present in *Ancognatha* Erichson, 1847 as a blunt or acute denticle (see Ramírez-Salinas et al. 2004; Neita-Moreno and Morón 2008). In Phileurini S_4_ is, if present, always smaller and triangular (Vanin et al. 1983; Dechambre and Lumaret 1986; Morelli [1991]; Ocampo and Morón 2004; Ratcliffe and Skelley 2011).

**Other larval characters:** According to Neita-Moreno and Orozco (2009), Oryctini larvae can be diagnosed by the combination of the following characters: cranium densely punctate, dark reddish brown; maxillary stridulatory teeth truncate; antennomere 4 with 2–15 dorsal sensory spots; tarsal claws with 2–4 long, stout setae; raster without palidia or septula. *Platyphileurus* larvae show all those characters. According to the larval characters this genus could belong to Oryctini, or possibly Dynastini (but these having rather rounded stridulatory teeth), but it is unlikely to belong to Cyclocephalini, Phileurini, or Pentodontini.

**Adult characters:** To exclude the possibility of *Platyphileurus* belonging to Oryctini, the adult mouthparts were examined. The mentum (labium) of *Platyphileurus* is triangular, basally broad, tightly tapered to a blunt, rounded, thin and slightly protruding tip (Fig. 29). The basis of the labial palps is almost visible, being slightly covered by the margin of the mentum only. This is different from the form of the mentum diagnostic for Phileurini, being broad and covering the basis of the labial palps completely. In fact, the mentum of *Platyphileurus* resembles broad menta of Oryctini or Pentodontini.

**Figures 26–30.**
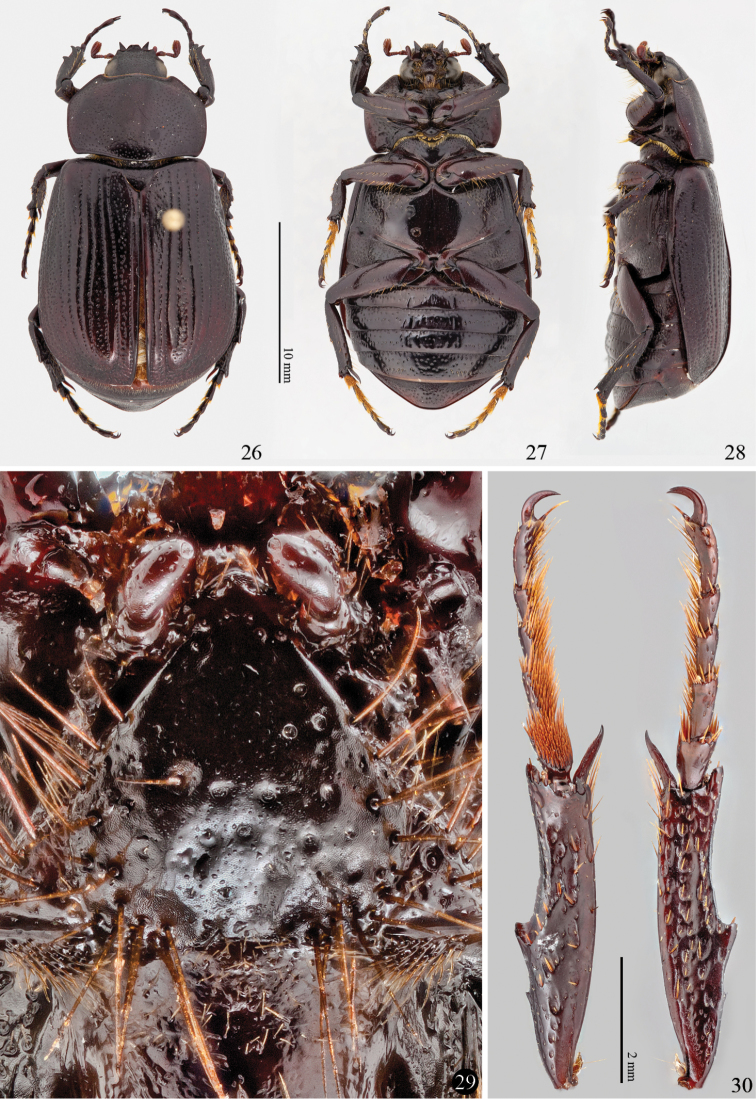
Imago of *Platyphileurus felscheanus* Ohaus, 1910. **26, 27 and 28** male habitus, dorsal, ventral and lateral, respectively **29** mentum DMNS ZE.20187 **30** hind leg ventral and dorsal view respectively DMNS ZE.20188. Photos: FF Albertoni (**26–28**), C Grinter (**29–30**).

**Figures 31–35.**
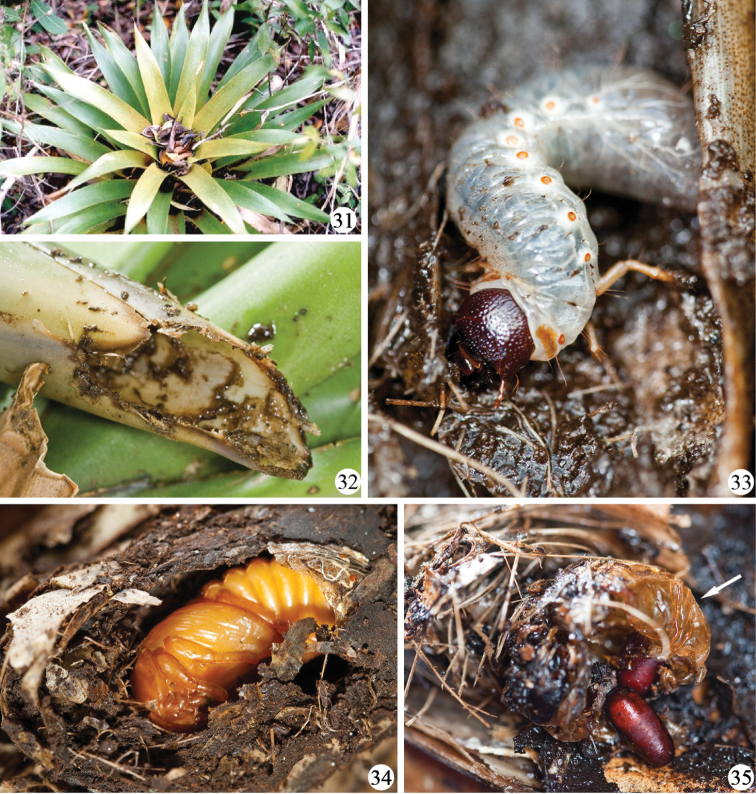
*Platyphileurus felscheanus* Ohaus, 1910, natural habitat: **31** bromeliad *Vriesea friburgensis* with leaf litter in open area of Atlantic Forest, Santa Catarina, Florianópolis **32** *Aechmea* sp., cylinder of the central leaves eaten by *Platyphileurus felscheanus* larva **33** larva on bromeliad leaf from the very center rosette **34** pupa in its pupal chamber built with pieces of leaves, twigs and humus **35** puparia of *Mystacella* sp. (Diptera: Tachinidae) and pupal exuvia of *Platyphileurus felscheanus* (arrow). Photo: A Zillikens (**31**), FF Albertoni (**32–35**).

**Figure 36–37.**
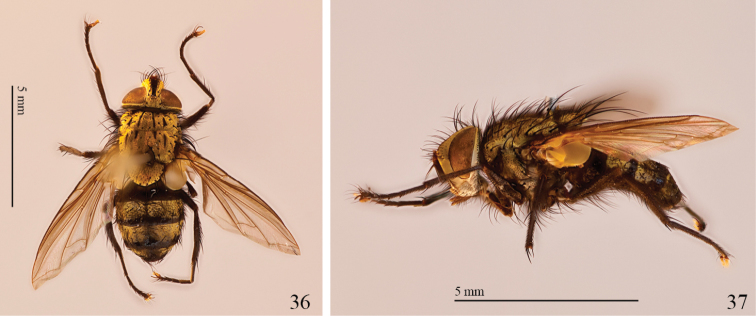
Imago of *Mystacella* sp., parasite of *Platyphileurus felscheanus*. **36** dorsal view **37** lateral view. Photos: FF Albertoni.

**Problems with the current tribal classification:** In the current, typological classification, Oryctini are separated from Pentodontini by one variable adult character, the apex of the hind tibia, of which Ratcliffe and Cave (2006: 189) noted: “We remain concerned that this single, sometimes variable (or transitional) character used to separate taxa at the tribal level is not reliable.” Likewise, Dynastini are separated from Oryctini by only two adult characters: a cylindrical first tarsomere of the hind legs (triangularly dilated in Oryctini and other tribes) and anterior legs in males more or less prolonged (no such dimorphism in Oryctini and other tribes) (Endrődi 1985; Ratcliffe and Cave 2006). Although Dynastini could well be monophyletic characterized by those two potential autapomorphies, it would leave the Oryctini without an autapomorphy in the current classification since the character separating it from Pentodontini, the crenulated or denticulate metatibial apex, is also present in Dynastini and possibly a synapomorphy of Oryctini and Dynastini. Without an autapomorphy, Oryctini cannot be diagnosed as a monophylum (cf. Hennig 1982: 93; Wheeler 2012: 41). Being entirely typological, the current tribal classification is likely to contain paraphyletic or even polyphyletic tribes.

In a caryological study Dutrillaux et al. (2013) found that *Augosoma* Burmeister, 1847, currently in Dynastini, might be closer to *Oryctes* Hellwig, 1798 (Oryctini) than to other Dynastini. The recent cladistic analysis of Dynastini by Rowland and Miller (2012) proposes *Augosoma* to be sister to the remaining analysed genera of the subtribe Dynastina, indicating its early branching within the Dynastini. This analysis cannot help clarifying the relationship between Dynastini and Oryctini (or Oryctini+Pentodontini) since representatives of the latter were not included. The relationship between Oryctini and Dynastini remains unresolved, but the recent studies indicate that Oryctini could be paraphyletic in respect to a - possibly polyphyletic - Dynastini.

**Tribal placement of*Platyphileurus*:** The apex of the hind tibia in *Platyphileurus* (Fig. 30), being (weakly) dentate and not truncate, would place this genus in Oryctini or Dynastini, not in Pentodontini in the current sense. This is supported by the number of sensilla on the larval antenna and the shape of the left larval mandible. The slightly broader first tarsomere of the hind legs, together with the small body size being unusual for Dynastini, indicates that it rather belongs to Oryctini than to Dynastini in the current sense. The shape of the adult mentum, the large number of sensilla on the larval antenna, and the strong S4 of the left larval mandible are not found in Phileurini. We accordingly propose the transfer of *Platyphileurus* from Phileurini to Oryctini. We consider the flat body of *Platyphileurus* a convergence with the body shape of Phileurini, likely related to the unique habitat among the tight bromeliad leaves, where the larva pupates and that enable adults hiding between their leaves.
